# Retraction: Swati Arora, Nagendra Verma. Exosomal microRNAs as potential biomarkers and therapeutic targets in corneal diseases. Molecular Vision 2024; 30:92-106

**Published:** 2024-08-29

This article was retracted by agreement among the authors and the journal's Editors-in-Chief.

Sincerely,

The Editors of Molecular Vision

